# The Delay of the Record

**Published:** 1875-08

**Authors:** 


*The Delay of the Record.*

Causes, which will in due time be freely explained, the RECORD has not been prompt in appearing on the days announced. These delays have been peculiarly annoying to all connected with the RECORD—especially the delay of the August number.

Having made new arrangements, the undersigned can safely promise that these vexatious delays shall not occur in the future.

The necessary numbers for the current year shall be pushed, and the volume completed at the usual time.

Correspondence having accumulated, letters still remain unanswered. This will be attended to as rapidly as possible. In the meantime, should subscribers and others fail to receive replies, we beg them to write again, in order to be sure that their letters have reached their destination. It is always a pleasure to attend to the wishes of our friends. P.

